# Microstructural Differences in Response of Thermoresistant (Ceramic) and Standard (Granite) Concretes on Heating. Studies Using SEM and Nonstandard Approaches to Microtomography and Mercury Intrusion Porosimetry Data

**DOI:** 10.3390/ma11071126

**Published:** 2018-07-02

**Authors:** Wojciech Franus, Anna Halicka, Krzysztof Lamorski, Grzegorz Jozefaciuk

**Affiliations:** 1 Department of Civil Engineering and Architecture, Lublin University of Technology, Nadbystrzycka 40, 20-618 Lublin, Poland; a.halicka@pollub.pl; 2 Department of Metrology and Modelling of Agrophysical Processes, Institute of Agrophysics, Doswiadczalna 4, 20-290 Lublin, Poland; k.lamorski@ipan.lublin.pl; 3 Department of Physical Chemistry of Porous Materials, Institute of Agrophysics, Doswiadczalna 4, 20-290 Lublin, Poland; jozefaci@ipan.lublin.pl

**Keywords:** concrete, high temperature, resistance, microstructure, sanitary ceramics, waste materials

## Abstract

The microstructure of concretes containing ceramic sanitary ware waste and granite aggregates was studied using scanning electron microscopy, mercury intrusion porosimetry and computer microtomography, before and after cyclic heating of the concretes to 1000 °C. All methods showed an increase in porosities in the concretes after heating. The proposed new approach to microtomography data analysis detected a much higher increase in the number of cracks in granite than in ceramic concrete after heating. This new approach to combining mercury intrusion and microtomography data showed that heating led to the narrowing of throats connecting smaller pore voids and a broadening of throats connecting larger pore voids, in both concretes.

## 1. Introduction

Reuse of non-biodegradable industrial waste, as well as materials coming from the demolition of various structures, has recently become a challenge for the material sciences. Ceramic materials, which are highly resistant against physical, biological, and chemical weathering, belong to this group. As a matter of good practice, recycling of ceramic waste most frequently involves incorporation of powdered or crushed ceramics into cement, mortar, or concrete, which provides an environmentally friendly way of reducing demand for natural aggregate and sand [1,2,3,4,5,6]. The addition of ceramic modifies the concrete structure. The interfacial zone (ITZ) between paste and ceramic aggregate, observable with scanning electron microscopy (SEM), becomes more compact, narrower, and less porous than that between paste and granite [7]. Better integration of the aggregate with concrete matrix improves mechanical behavior and produces positive effects on durability. Porosimetry and scanning electron microscopy has proved that concrete porosity increases with a higher percentage of sanitary ceramic ware aggregate, simultaneously reducing macropores larger than 0.05 µm and increasing capillary pores [7,8]. Canbaz [9] observed that ceramic-filled concretes are resistant against high temperatures. He found that high temperature had no negative effect on the compressive and flexural strength of concrete with 100% sanitary ceramic ware aggregate, whereas if the amount of ceramic was smaller, a 30% strength reduction occurred. Halicka et al. [10] found that sanitary ceramic ware waste aggregate and high alumina cement may be satisfactorily used for manufacturing concrete elements intended to work in high temperatures. After being heated to 1000 °C, concrete with ceramic sanitary ware aggregate preserved its form and high strength, whereas the opposite was the case for sand and granite concrete, and granite concrete.

High temperature is well known to be seriously damaging to concrete micro- and meso-structure, in that it brings in a generalized mechanical decay of the concrete, and even detrimental effects at the structural level [11]. Usually, heating leads to a drastic decrease in concrete strength. Poon et al. [12] found that heating to 600 °C caused up to 40% and to 800 °C up to 80% decrease in compressive strength, respectively. Handoo et al. [13] observed total deterioration of Portland concrete above 900 °C. Although mechanisms of structural changes of various concretes after heating have been studied in many papers [14,15,16,17,18,19,20], the authors found no results concerning microstructural response of highly temperature-resistant ceramic concretes on heating, which is the primary aim of the present paper. We expected differences in thermal resistance of the concretes to be reflected in microstructural changes.

Concrete microstructure is most commonly studied using mercury porosimetry (MIP) [21,22,23,24], computer microtomography (MCT) [25,26,27] and scanning electron microscopy (SEM) [28,29,30,31]. We also applied the above methods; however, we tried to extend the interpretative possibilities of their results. It is commonly accepted that MIP can estimate not pore sizes but sizes of pore entrances (necks or throats) connecting pores of ink-bottle type [32,33]. To date only applications of mathematical pore-shape models serve to relate pore bodies to their throat sizes [34]. We joined MIP and MCT data to do this. Moreover, we tried to estimate an occurrence of internal cracks in concretes from pore-shape factors derived from MCT images, which we had also not found in the literature. The second aim of this paper is see whether the above nonstandard ways of data interpretation may broaden the applicability of both methods.

## 2. Materials and Methods

### 2.1. Materials and Samples Preparation

Ceramic waste taken from a dump at the Cersanit factory (Krasnystaw, Poland) was used. It contained mainly mullite and some quartz, cristobalite and calcite in crystal phases. The amorphous substance was aluminosilicate glaze. The waste was crushed using a jaw crusher and passed through 8 mm and 4 mm sieves. Particles of 0–4 and 4–8 mm were collected and mixed at 10:4 ratio. Fineness-grading modulus (FM) calculated in accordance to EN 12620:2002 standard was equal to 4.51 and the amount of finest particle (<0.063 mm) was equal 0.37%. The chemical composition of the ceramics measured by EDS by Halicka et al. [10] was 67.63% SiO_2_, 24.05% Al_2_O_3_, 3.0% K_2_O, 2.78% NiO, 1.25% Na_2_O, 0.55% Fe_2_O_3_, 0.37% Mo_2_O_3_ and 0.36% MgO. As a reference material a granite was prepared, with a mixture of 0–4 and 4–8 mm granite fractions at 10:4 ratio, admixed with 0.37% of a fine sand (<0.063 mm) to have similar granulometric composition as the ceramic granulate.

Both above materials and high alumina cement (Górkal 70, Trzebinia, Poland) containing more than 70% of Al_2_O_3_ were used to produce concretes of the following composition: aggregate 1387.9 kg/m^3^, water 201.4 kg/m^3^ and cement 493.4 kg/m^3^. Molded concrete prisms of 40 mm × 40 mm × 160 mm and conditioned for four weeks were preheated twice at 150 °C for 36 h, once at 550 °C for 7 h and then subjected to five 1000 °C heating and 20 °C cooling cycles, as described in detail in Reference [10].

The studied materials are abbreviated further by the letters g (granite), c (ceramics), C (concrete) and the applied heating by the letter T. For example, CgT means heated concrete made from granite.

Compressive and flexure-strength tests of the prismatic specimens of the concretes studied in the present paper performed by Halicka et al. [10] certified much better thermal resistance of the ceramic than the granite concrete.

### 2.2. Density and Porosity Estimation

Particle density (PD) [g/cm^3^] of the studied materials was estimated from their volumes (measured by immersion in mercury) and masses (weighing), while their solid-phase densities (SPD) [g/cm^3^] were measured by helium pycnometry using AccuPyc II 1340 provided by Micromeritics (Aachen, Germany). From the above data the total porosities of the particle was calculated.

### 2.3. SEM Analysis

The SEM images of the tested concretes were taken using a FEI Quanta 250 FEG microscope. The broken concrete surfaces were analyzed. The samples were stuck onto a carbon holder with a carbon glue and sprayed with around 50 nm graphite layer. The images were analyzed visually. Each imaged region was selected to contain both the aggregates and the cement phase.

### 2.4. X-ray Microtomography Studies

X-ray computational microtomography (MCT) was applied in two replicates for 3D scanning of the studied materials using a General Electric Nanotom 180S device (Wunstorf, Germany), which allowed for 2 μm voxel size in 3D representation of the samples. Due to high scan resolution and possible impact of the heat-induced sample shape changes on image registration process, samples were scanned in two-steps. First, a short (30 min) pre-scan took place, then the proper scan was started. The reason for the pre-scan was to heat up the sample holder and the sample itself to prevent further shape changes. For each specimen, 1200 2D images were collected for the full angle sample rotation. Registered 2D images were subject to 3D reconstruction using the DatosX 2.0 software (Rubrik Inc., Palo Alto, CA, USA). As a result, 3D sample representations were recovered at a 16-bit gray-level volume. Then the image analysis, processing and visualization was performed using VG Studio 2.0 (Volume Graphics, Heidelberg, Germany) and Avizo 9 (FEI, 5350 NE Dawson Creek Drive, Hillsboro, OR, USA) software. The initial image-processing procedure consisted of: Region of interest (ROI) selection and 3D median filtering with the kernel diameter of 3 px. The scanned samples were cubic in shape, with about 5 mm edge length; however, the smaller cylindrical ROI, with 4 mm diameter and 3.2 mm height, was analyzed. The image was then thresholded using an iterative IsoData algorithm. After thresholding, the pore space was distinguished from solid phase within the accuracy that µCT scan resolution allowed. Then a 3D watershed algorithm was used, followed by labeling procedure. As a result, the set of distinguished individual pores was generated for which, after the label analysis step, individual pore volumes, surface areas, and shape factors were calculated (Avizo software). Total porosity and volume of individual voids were determined by counting the voxels belonging to the pores. Fractal dimension of the pore system was determined for thresholded images of the scanned specimens using a box-counting algorithm (Fiji software).

X-ray inspection showed that the pore space of the cement phase consists of two types of pores: elongated shell-like pores (cracks) occurring at the borders of filler grains and the cement, and spherical pores localized in the medium of the material. Many noninvasive techniques for detecting concrete microfracture have been used [35]. Here, we propose an alternative method for distinguishing the cracks from other pores by analyzing differences in pore shapes in the scanned concretes.

The criterion for distinguishing between cracks and spherical pores was the spherical shape parameter (SSP) defended as:

SSP = S_vox_ D_eq_/V_vox_, (1)

where S_vox_ is a pore surface, V_vox_ is a pore volume and D_eq_ is the equivalent diameter of a pore. All of the components of this equation were determined based on voxelized representation of the pores. The SSP parameter has a minimum value of 6 for ideal spherical shape. The pores of SSP values higher or equal to 15 were counted as cracks. Since for very small pores the cracks and pore connections are hardly distinguishable, the discrimination between cracks and pores was performed for pores larger than 2.5 × 10^−5^ mm^3^ (after labeling, the pores with smaller volumes were filtered out).

### 2.5. Mercury Intrusion Porosimetry Studies

Mercury intrusion porosimetry (MIP) tests were performed for the studied materials in triplicate for pressures ranging from ca. 0.1 to 200 MPa (pore radii from ca. 10.0 to 3.8 × 10^−3^ µm) using Micromeritics’ AutoPore IV 9500 unit (Norcross, GA, USA). The studied concrete samples included two that had been scanned by MCT, and one extra. The initial granite and ceramics aggregates measured had volumes of around 0.15 cm^3^. We did our best to make these volumes represent the average granulometric composition of the materials. Prior to measurement, all specimens were heated overnight at 105 °C and then degassed under vacuum, which is the standard procedure in MIP analysis. The intrusion volumes were measured at stepwise increasing pressures, allowing all specimens to be equilibrated at each pressure step. The maximum deviations between the mercury intrusion volumes were not higher than 2.4%, and they occurred mainly at low pressures (largest pores). The intrusion pressure was translated on equivalent pore throat radius R [m] following the Washburn equation:

P = −A σ_m_ cosα_m_/R, (2)

where σ_m_ is the mercury surface tension (0.485 N m^−1^), α_m_ is the mercury/solid contact angle, and A is the shape factor (equal to 2 for the assumed capillary pores). The value of the contact angle was taken as 140° for granite and ceramics, and as 130° for the concretes, according to Groena et al. [36], who demonstrated that the contact angle of mercury on most oxidic materials presented a contact angle close to 140° and on cement-like materials close to 130°.

The volume of mercury V [m^3^kg^−1^] intruded at a given pressure P [Pa] gave the pore volume that could be accessed. It is accepted [37] that two main types of pore filling exist: (1) the main intrusion of mercury into the interconnected pore networks of a particle body, and (2) artifacts related to the invasion of mercury into relatively large pores between the sample particles and the pores located at the particles’ surfaces. Therefore, the initial part of the mercury injection curve was associated with “surface defects” and the next part, beginning after a so-called penetration threshold (PT), with mercury penetration into the pores inside the particles. The penetration thresholds were approximated by the pore radii at which the second derivative of pore volume vs. log radius equals zero [38]:

d^2^V/d(logR)^2^ = 0, (3)

The parts below PTs were eliminated from the original mercury intrusion curves.

Knowing the dependence of corrected pore volume V vs. R, a normalized pore throat size distribution, χ(R), was calculated and expressed in the logarithmic scale [39]:

χ(R) = 1/V_max_ dV/dlogR, (4)

Knowing χ(R), the average pore throat radius, R_av_, was calculated from:

R_av_ = ∫R χ(R) dR, (5)

If a range of pore sizes wherein the pore volume depends on a power of the pore radius could be found, this was interpreted in terms of pore surface fractal scaling. In this case the dependence of log(dV/dR) against logR was plotted, and from the slope of its linear part the fractal dimension of pore surface D was derived as [40]:

Ds = 2 − slope,(6)

To define linear range of fractality, the procedure of Yokoya et al. [41] was applied. According to this procedure the measure of the linearity L for the set of the points in a x–y plane is:

L = (4σ^2^_xy_ + (σ_yy_ − σ_xx_)^2^)^1/2^ (σ_yy_ + σ_xx_)^−1^, (7)

where σ_xx_, σ_yy_, and σ_xy_ are the variances of x-coordinates, y-coordinates, and the covariance between x- and y-coordinate sets, respectively.

The L-value falls between 0 (for uncorrelated and random points) and 1 (for points on a straight line). To separate linearity range, the value of L is computed for the first three points, then for the first four, five and so on until the value of L increases. The end of the linearity range is to be found at the points after which the value of L begins to decrease. From the estimated linearity range the two first and/or two last points were rejected if this caused an increase in the linear regression coefficient between the considered data.

The apparent solid-phase skeletal densities of the samples, SSD_app_ (which are lower than true skeletal densities due to the residence of the finest pores in the solid phase, which are not filled by mercury at its highest pressure), and total surface of MIP available pores, S(MIP), were calculated by the porosimetric data analysis program provided by the equipment manufacturer.

Since concretes are composed from particulate components, the pores in concrete occur around contact points between these components, and so the pores have larger chambers (voids) joined by narrower throats. Accessibility of the voids through the throats is particularly important for MIP measurements because the throats control mercury intrusion into the voids. Therefore, Dullien and Dhawan [42] postulated that in granular media the dependence of the total volume (throat + void) accessible through the throats of hydraulic radii, r_throat_, can be estimated by MIP. Thus, MIP gives the throat size distribution. With a high probability one can postulate that MCT can identify only pore voids, thus it gives the void size, r_void_, distribution. We applied both of the above postulates to propose a new approach to (roughly) relate the throat radii to the void radii by combining MIP and MT data [43], which as yet has not been used for concrete characterization. To do this we calculated the average MCT and MIP pore radii within the same sub-ranges (fractions) of MCT and MIP pore volumes. Ten equal sub-ranges (0–0.1, 0.1–0.2, 0.2–0.3, etc.) of pore volumes were selected. Having calculated the average radius of the void (MCT) and of its throat (MIP) within the same sub-range, the r_void_ versus r_throat_ dependence was constructed.

## 3. Results and Discussion

**Figure 1:**
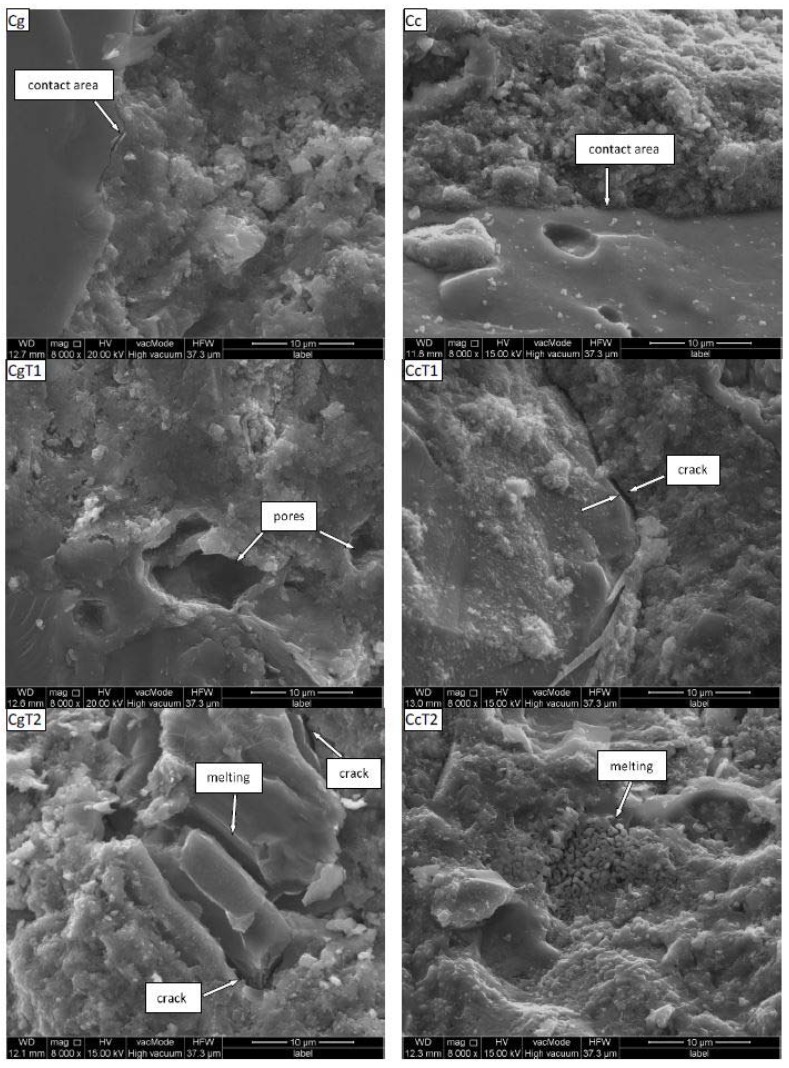
Representative SEM images of the surfaces of the broken concretes studied. Abbreviations: C–concrete, c–ceramics, g–granite and T–heated.

Representative SEM images of the studied concretes are shown in Figure 1. In both concretes before heating a good contact of the aggregates with the cement phase is seen, with almost no leaks and cracks [44]. Thermal treatment alters the microstructure of both concretes. The granite-based concrete exhibits numerous irregular cracks and pores (CgT1), and fragments of melting of nonresistant components (CgT2). In the ceramic-based concrete cracks and leaks occur occasionally (CcT1). On the ceramic surface changes in phase composition and internal structure were not observed (CcT2).

Wang et al. [45] studied microcracking of two concretes at different temperatures via in situ SEM observations, finding that microcracks generally initiate around the boundary of sands and that the failures are mainly brittle, even at high temperature. They explained the original crack propagation at the boundaries of aggregate particles by the boundary effect because the shrinkage ratios of both aggregate particles and intermixed are different. Our observations that the ceramic concrete undergoes less alteration after heating suggest that the thermal expansion of the porous ceramic aggregates exerts less force on the surrounding cement phase. Possibly, within the porous body of the ceramic aggregate, there is enough space for the expansion of the heated ceramic phase, contrary to the granite phase which should be much more compact.

**Figure 2:**
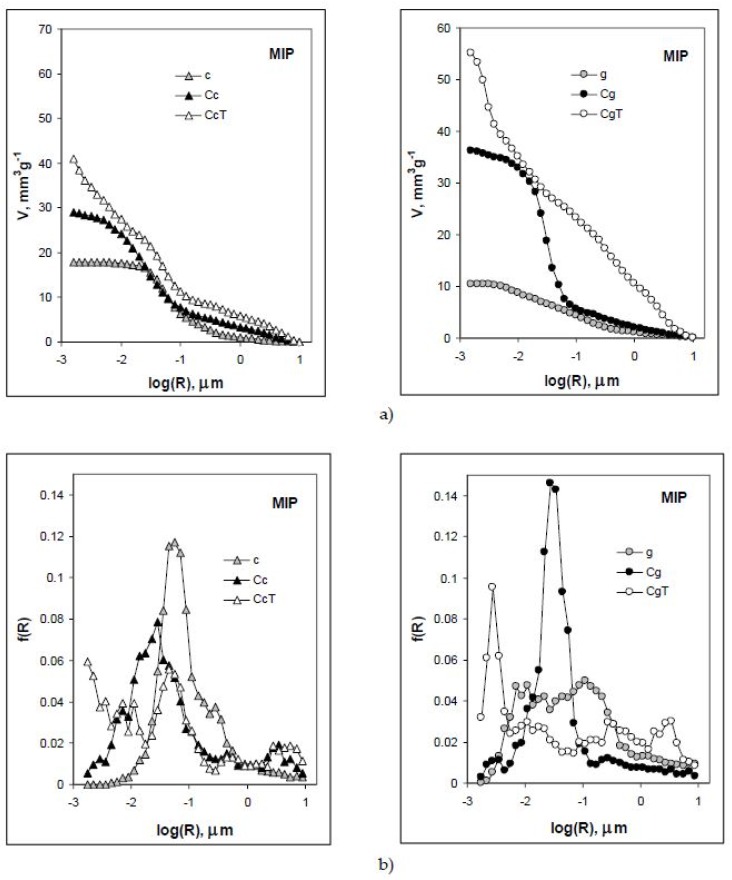
Mercury intrusion (MIP) pore volume vs. pore throat radius curves (**a**) and pore throat size distribution functions (**b**) for the studied materials. Abbreviations: c–ceramics, g–granite, C–concrete and T–heated.

Mercury intrusion porosimetry curves relating the volumes of internal pores (voids + throats) and radii of pore throats for the studied specimens are shown in Figure 2a. The curves presented are averaged from three replicates. It is important to note that the penetration thresholds for most of the samples are located around R = 1 µm. The only exception is the spalled concrete made from granite (CgT) for which PT occurs around R = 3.161 µm. Figure 2b shows pore throat size distributions. The ceramics have higher porosity than the granite. The concretes have higher porosities than their fillers. The heating process leads to increases in concrete porosities, which is much more pronounced for the granite-based material, particularly in the range of relatively large pores (10–0.1 µm).

As shown in Figure 2b, the nonheated concretes have more uniform (practically unimodal) pore throat size distribution functions than the heated ones. Thermal treatment leads to an increase in the finest and the largest pore throat fractions, at the expense of medium-size throats in both concretes. The relative increase in small and large throat fractions is markedly higher in granite-filled concrete.

The above results indicating much larger effect of heating on pore sizes and volumes of the granite concrete are consistent with SEM observations.

**Figure 3:**
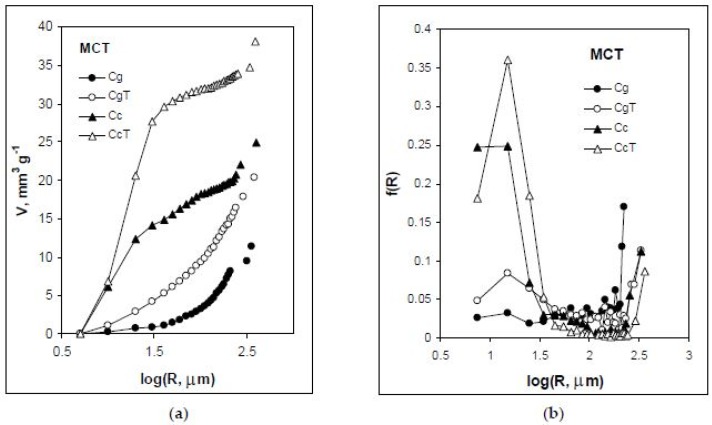
Microtomography (MCT) pore volume vs. pore radius curves (**a**) and pore size distribution functions (**b**) for the studied concretes. Abbreviations: c–ceramics, g–granite, C–concrete and T–heated.

Figure 3 shows pore volume vs. pore radius curves and pore size distribution functions for the studied concretes derived from microtomography (MCT) data. The granite and ceramics contained no MCT-detectable pores. Similarly, as for the MIP-range pores, the heating process leads to an increase in concrete porosities and an increase in the finer pore fractions in MCT-detectable pores. Relatively, this increase is again higher in granite concrete.

As a result of the water evaporation and the chemical changes of hydration products, elevation of temperature increases porosity and pore size of cement and concrete [8,9]. The coarsening of the pore structure is mainly responsible for the reduction of the mechanical properties [46]. The studied concretes exhibit this common behavior. The ceramic concrete has higher amounts of around 1 µ pores. It is possible that pores of that size create some additional space for thermally expanded solid particles, supporting the counteraction of internal ceramic pores against thermal expansion of the whole concrete. This could be a reason for the higher thermal resistance of the ceramic concrete.

**Figure 4:**
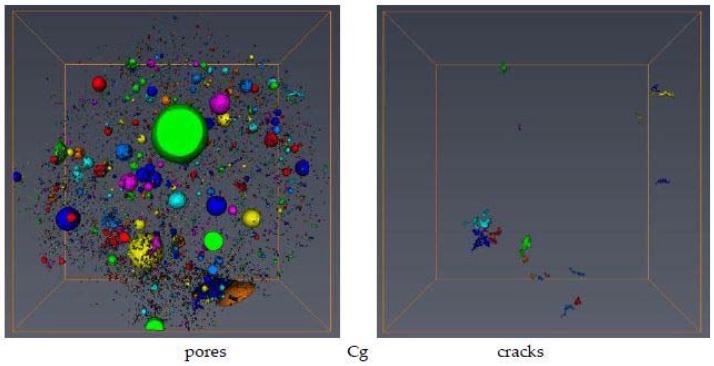

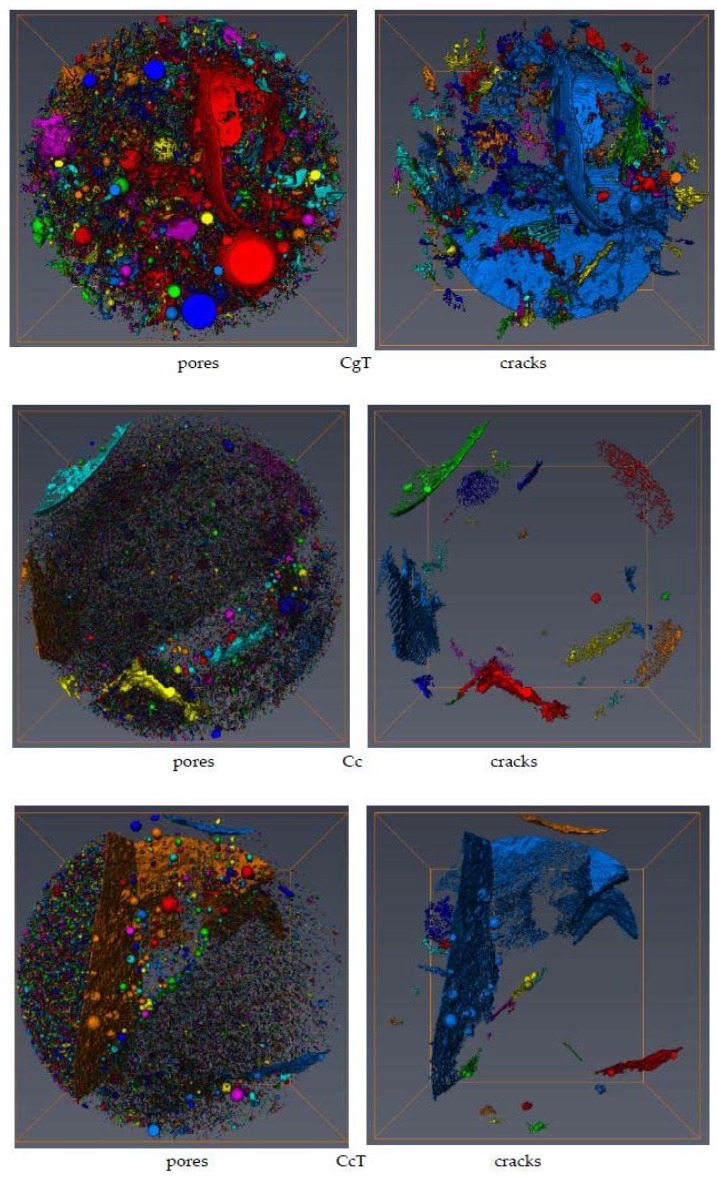
Microtomography (MCT) top view of the samples (diameter 4 mm) for the studied concretes showing all pores (**left**) and the separate cracks (**right**). Different colors mark individual spaces (pores or cracks) to better guide the eye. Abbreviations: C–concrete, g–granite, c–ceramics and T–heated.

Figure 4 shows representative microtomography scans of the studied concretes before and after heating. The cracks distinguished by their elongation factor are shown separately.

Thermal treatment seriously damages the microstructure of the granite concrete, as is seen from sharp increase in the number of pores and cracks, whereas the ceramic concrete microstructure survives heating much better.

Table 1 compiles porosity parameters of the studied samples derived from the applied methods.

True particle densities of the studied materials are as a rule higher than their counterparts measured by mercury intrusion porosimetry, and the same holds for solid-phase densities, a result of the presence of residual pores not invaded by mercury at its highest pressure. Granite aggregates and the concrete manufactured from them have higher densities than the ceramic aggregates and concrete. Pore volumes and porosities measured by MIP are significantly higher than those measured by MCT. Surprisingly, the porosity calculated from particle density (mass estimated from weighing and volume from mercury immersion) and solid-phase density (measured by helium pycnometry) is in most cases slightly lower than that measured by MIP, despite the fact that it should be higher. The reason for this is not clear to us. Markedly smaller changes in pore volumes and porosities due to heating are noted for concrete manufactured from ceramics than for concrete manufactured from granite. The volumes of pores located inside particles of the studied materials (internal pores) are obviously lower than the total amount of the MIP pores and they are still much higher than the MCT-pore volumes. A decrease in average pore radius was observed for MCT pores, whereas no evident trend was noted for MIP pores. However, an increase in total pore area measured by MIP was observed in both concretes after heating. Microtomography detected an increase in pore complexity after concrete heating (higher fractal dimensions), whereas mercury intrusion porosimetry detected an opposite trend. The number of concrete cracks increases after heating. For the granite concrete around a 60-fold increase was noted, while for the ceramics concrete the number of cracks increased only two-fold.

**Figure 5:**
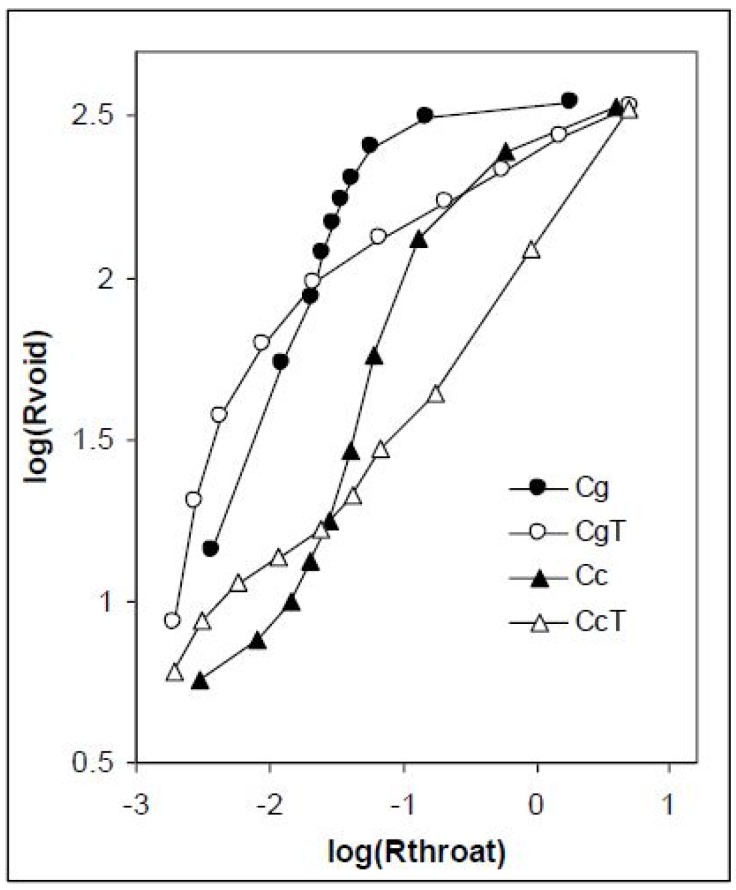
Dependence of pore void to pore throat radii for the studied concretes before and after heating. Data in logarithmic scales are depicted.

The dependencies of the radii of the pore voids (MCT) to the pore throats (MIP) are shown in Figure 5.

Heating of both concretes causes structural changes in pore buildup. In the range of narrower throats (up to around 0.03 µm, logR ≈ −1.5) the voids of equal sizes are more accessible through narrower throats in heated than in nonheated concretes, whereas in the range of larger throats the situation is the opposite. It seems interesting that in both concretes the same trends of structural changes after heating occur exactly at the same throat radius (0.03 µm). We think that this is related to the cement used, and that different cements may exhibit different behaviors. This hypothesis is worth checking, which we plan to do in the near future.

Because the methods proposed give deeper insight into concrete microstructure and its reaction on heating, we think that they may be applied to further explore different concrete properties under various environmental and anthropogenic factors. Our observations that higher thermal resistance of concrete increases with an increase in its porosity seems very interesting for us. We think that within a more porous body there is more free space for thermally expanded and/or deformed phases. It is worth checking how various porosities and different sizes of pores affect the thermal resistance of concretes of the same chemical composition.

## 4. Conclusions

Scanning electron microscopy (SEM), mercury intrusion porosimetry (MIP) and microtomography (MCT) were applied to describe heating-induced differences in the microstructure of granite and waste ceramics concretes in a broad range of scales. A heating-induced increase in total porosities measured by all methods was accompanied by a decrease in particle density. Formation of new, very fine pore throats and an increase in large throats was observed by MIP. Additionally, an increase in small (around 1 µm) pores was revealed by MCT. The fractal dimension determined from MCT increased after heating, indicating an increase in large-pore complexity, and when measured by MIP this decreased, indicating a decrease in finer pore throat complexity. No evident trends of changes in average radii of pore throats were observed by MIP, whereas an increase in MCT pores was detected after heating.

Using computer elaboration of microtomography scans, we detected the formation of cracks in the studied concretes. In more temperature-resistant concrete manufactured from ceramics, heating only doubled the number of cracks, whereas in standard non-temperature-resistant concrete manufactured from granite aggregates, heating increased the number of cracks by around 60 times.

By combining microtomography and mercury intrusion data we estimated a rough dependence between pore voids (MCT) and radii of throats (MIP). In both concretes, heating caused narrowing of the throats leading to smaller voids and broadening of the throats leading to larger voids.

## Figures and Tables

**Table 1:** Porosity characteristics of the studied materials.

Parameter	Unit	g	Cg	CgT	c	Cc	CcT
Particle Density (mass and volume)	g cm^−3^	2.63	2.38	2.28	2.40	2.34	2.24
Particle Density (MIP)	g cm^−3^	2.56	2.35	2.22	2.35	2.31	2.24
Solid-Phase Density (He pycnometry)	g cm^−3^	2.74	2.62	2.63	2.55	2.51	2.50
Solid-Phase Density (MIP)	g cm^−3^	2.71	2.60	2.58	2.53	2.49	2.50
Pore Volume (MIP)	mm^3^ g^−1^	15.0	39.7	61.6	21.2	31.9	46.4
Pore Volume (MCT)	mm^3^ g^−1^	n.d.	11.4	20.4	n.d.	24.9	38.0
Pore volume (Part. Dens./He pyc.)	mm^3^ g^−1^	15.3	38.5	58.4	24.5	28.9	46.4
Internal Pores Volume (MIP)	mm^3^ g^−1^	10.4	36.1	55.0	17.8	28.9	41.0
Porosity (MIP)	% *v*/*v*	5.47	9.33	13.69	7.12	7.37	10.39
Porosity (MCT)	% *v*/*v*	n.d.	2.68	4.53	n.d.	5.75	8.51
Porosity (Part. Dens./He pyc.)	% *v*/*v*	4.01	9.16	13.31	5.88	6.77	10.40
Average Pore Radius (MIP)	μm	0.50	0.08	0.11	0.25	0.09	0.08
Average Radius of Internal Pores (MIP)	μm	0.073	0.044	0.057	0.096	0.056	0.047
Average Pore Radius (MCT)	μm	n.d.	118.7	93.7	n.d.	34.2	26.5
Total Pore Area (MIP)	m^2^ g^−1^	1.39	3.49	14.04	0.91	3.25	10.33
Fractal dimension (MCT)	-	n.d.	1.85	2.17	n.d.	2.45	2.61
Fractal dimension (MIP)	-	3.35	3.28	3.20	3.55	3.26	3.10
Volume of cracks (MCT)	mm^3^ g^−1^	n.d.	0.12	7.38	n.d.	1.27	2.99

Abbreviations: MIP mercury intrusion porosimetry, MCT microtomography, c–ceramics, g–granite, C–concrete and T–heated.
